# An electrophysiological investigation into ‘Bagels’: a song recorded to reduce subclinical anxiety in youth

**DOI:** 10.3389/fnins.2025.1640674

**Published:** 2025-10-07

**Authors:** Daniel Shepherd, Mangor Pedersen, Geet Vashista, Amy Kercher, Michael J. Hautus

**Affiliations:** ^1^Psychophysiology Laboratory, Department of Psychology and Neuroscience, Auckland University of Technology, Auckland, New Zealand; ^2^Psychophysics Laboratory, School of Psychology, University of Auckland, Auckland, New Zealand

**Keywords:** music medicine, anxiety, youth, brain connectivity, skin conductance, heart rate

## Abstract

**Introduction:**

Receptive Music Therapy allows individuals with sub-clinical anxiety levels to self-medicate when and where they choose, but the effectiveness of self-administered ‘music medicine’ to enhance psychological well-being is still being investigated. The current study reports on a song (‘Bagels’) designed to alleviate mild anxiety in adolescents and young adults.

**Methods:**

A laboratory study was conducted to examine the effect of Bagels on brain states, and upon both subjective and objective measures of state anxiety. Measures of skin conductance and heart rate (HR), and 64-channel Electroencephalography (EEG) were obtained from 30 young adults as they listened to six songs contrasting affective properties. Subjective measures included ratings of a song’s pleasantness, arousal, dominance, and likability, as well as estimates of state anxiety obtained immediately after listening to them.

**Results:**

Preliminary analyses revealed that the six songs differed significantly in terms of affective ratings, with Bagels rated as more pleasant and less arousing, and having lower state anxiety ratings at its terminus. EEG alpha connectivity was also lowest for the song Bagels, particularly in the brain’s frontal lobes. Similarly, Bagels was associated with lower physiological arousal, reflecting less arousal and greater calmness.

**Discussion:**

Combined, the analysis suggest that Bagels has the potential to be an effective digital anxiolytic. Discussion around the promise of music medicine and aspects of its management are presented, along with avenues of further inquiry.

## Introduction

The deliberate use of sound to enhance psychological well-being and nurture positive emotions describes the practice of music therapy, an evidence-based treatment approach first promoted in the scientific literature by Gardner in the 1940s (Gardner, 1944). Traditionally, music therapy involved patients playing, in solitude or in groups, a musical instrument purposely selected to enhance their recovery. Later, with the arrival of equipment capable of recording and reproducing sound, the potential for patients to consume music for curative purposes emerged. Tang et al. (2020) discriminate between traditional music therapies, as administered by a trained practitioner (i.e., active music therapy), and music medicine (i.e., receptive music therapy), in which individuals self-administer doses of pre-recorded music to reduce negative emotions. In modern times, many have access to technology that affords the personal selection and consumption of sounds or music, and the marketing of sounds as acoustic relaxants by the so-called “self-help” industry is common (Hautus et al., 2021). However, despite the potential of music to allow individuals to self-regulate emotions, scrutiny of the available self-administered sonic medicines reveals scant scientific evidence backing up the claims of their merchants (Tan et al., 2012).

There are indications in the literature that music can be used to reduce depression (Tang et al., 2020) and anxiety (Harney et al., 2023). Meta-analyses conducted on music interventions undertaken in medical or therapeutic settings have indicated that traditional music therapy can significantly reduce negative emotions caused by stress and anxiety, irrespective of whether subjective or objective measures are used (de Witte et al., 2020). Anxiety is a significant and growing issue for young people, with meta-analyses reporting that 31% of adolescents experienced elevated anxiety during the recent COVID-19 pandemic (Deng et al., 2023). When individuals are not equipped with the means to regulate or reduce anxiety, it can cause substantial distress and interference, and is associated with comorbid psychological conditions such as depression, suicidal behavior and substance misuse (Woodward and Fergusson, 2001). While current treatment guidelines recommend cognitive-behavioral therapy (CBT) and selective serotonin reuptake inhibitor (SSRI) medication, they also emphasize an urgent need for further intervention approaches (Walter et al., 2020).

Finding innovative ways to support people with feelings of anxiety is a priority for clinicians and researchers, and studies have indicated that music medicine can be used to boost self-esteem, reduce stress, anxiety, or depression, and regulate emotion (Harney et al., 2023; Saifman et al., 2023; Shepherd et al., 2023). The mechanism by which music can potentially regulate emotions centers on the Autonomic Nervous System, with the Stress Reduction Theory (SRT) stating that sound can induce positive emotions and attenuate the body’s stress response. The Sympathetic Nervous System regulates the stress response, which is central to the body’s fight-or-flight response. The SRT argues that some sounds can reduce negative emotions and restore the dominance of the body’s rest-and-digest system, known as the Parasympathetic Nervous System. This theory echoes the ‘Undoing Hypothesis’ (Fredrickson et al., 2000), which states that positive emotions facilitate the ‘undoing’ of sympathetic arousal and feelings of stress. At the cognitive level, the Attention Recovery Theory (ART) argues that natural soundscapes can facilitate both attentional processes and cognitive performance (Kaplan and Kaplan, 1989), with emerging evidence suggesting that music possesses the ability to enhance cognition (Dovorany et al., 2023).

Quantifying how music can potentially manipulate psychological states requires a stimulus–response approach. Emotional states are psychological processes that are intrinsically difficult to measure directly, and in the literature both subjective and objective measurement approaches are commonly encountered. As emotions can be considered experiential it makes sense to simply ask an individual about their current state, allowing self-referential measures to be obtained. These subjective judgments are usually gathered using single questions or multi-item inventories, however, they rely upon the honesty and insight of the individual. As an alternative to self-report data, objective measures are made feasible by the fact that music activates the Autonomic Nervous System, and that emotions elicited by music are as strong as any other emotion (Rickard, 2004). Common objective measures of emotion include skin conductance, heart rate, blood pressure, and respiration.

One emerging avenue of investigation has been to look at brain communication during music listening. The necessity of multiple cortical area recruitment during music listening has led researchers to utilize connectivity and graph theory to study the brain (e.g., Karmonik et al., 2016; Wu et al., 2012). Here, findings suggest that music listening affects multiple wave bands, notably alpha frequencies, and corresponds with bilateral alterations in the frontotemporal electroencephalographic (EEG) profile (Altenmüller et al., 2002). Furthermore, EEG alpha oscillatory activity is pertinent to audio processing (Weisz et al., 2011), and listening to music is linked to increased EEG alpha connectivity compared with listening to random noise. Of direct relevance to the current study, higher EEG alpha connectivity correlates with anxiety in childhood and adolescence (Schmidt et al., 2022).

There is ongoing debate as to whether there are specific attributes of music that are more potent in regulating emotions, such as music tempo, the absence or presence of lyrics, and musical key, or whether it is the subjective response an individual has to the music that determines its effectiveness. Central to this debate is that prescribed music may be less beneficial than self-selected music as it may not be either familiar or liked by the listener. Classical music, long considered the gold standard of anxiolytics (Adiasto et al., 2022), may not be effective with younger people who prefer contemporary rock and pop genres, compared to older adults who prefer more relaxing genres such as classical or jazz (Davies et al., 2022). However, the notion that better outcomes are achieved when individuals self-medicate and select their own music has not been supported in meta-analyses (Harney et al., 2023). While some studies have reported a significant effect of self- versus researcher-selected music (Juslin et al., 2008) others have argued that the psychophysical properties of the music have greater agency than preference (Tan et al., 2012; Elliott et al., 2011; Iwanaga and Moroki, 1999).

In contemporary music and health research, Adiasto et al. (2022) observed that the majority of studies have been undertaken in either medical or therapeutic settings, using inpatients whose treatment-related stress (e.g., pregnancy and labor) or anxiety (e.g., fear of dying or an upcoming operation) is being managed by a trained music therapist. As others before them (e.g., Elliott et al., 2011) they opined such results couldn’t be generalized to non-clinical out-patient populations exposed to stressors or experience anxiety in their daily lives. Thus, while music has demonstrated a consistent stress-reducing and anxiolytic effect within medical environments, the research into non-clinical populations – who are more likely to use music medicine instead of music therapy – is both equivocal and lighter in volume. Further research into music medicine is warranted, particularly in young adults in which anxiety symptoms often begin to emerge in the face of life transitions, and because interest in music peaks between mid-to-late adolescence and the beginnings of adulthood (Davies et al., 2022).

The resource-intensive nature of psychotherapy, or potential for medication side effects or treatment non-compliance, has created a demand for further innovation and new approaches to anxiety interventions. There is emerging evidence that self-administered music, freely available from digital sources, may effectively regulate state anxiety for young people. Previous laboratory-based research supported the potential for Weightless, a song specifically designed to calm and facilitate parasympathetic activity, to act as an anxiolytic (Shepherd et al., 2023). Labbé et al. (2007) noted that music is a sonic relaxant frequently used by young people, who may compile ‘playlists’ of songs whose common function is to restore positive emotions and allow them to ‘chill’. In a study of undergraduate students, Elliott et al. (2011) endorsed music as an ‘on the spot’ relaxation technique able to release superfluous tension. On the back of emerging evidence, the current study involved the composition of a new piece of music, Bagels, developed with the help of youth mental health providers as a tool for adolescents and young adults to self-manage acute, but subclinical, anxiety.

### The current study

The ability of music to act as a sonic medicine has long been of interest in the literature, with studies focusing on music’s ability to reduce negative emotions such as anxiety, depression, and stress. The evidence indicating that music alleviates self-reported anxiety is becoming more abundant (Harney et al., 2023), though some have been critical of studies using ‘off-the-shelf’ songs with little insight or reference into the psychophysical relationship between music and either relaxation (Tan et al., 2012) or music and anxiety reduction (Elliott et al., 2011). Thus, for the current study we sought to create a novel music-based option enabling young people to self-manage mild anxiety symptoms and reduce feelings of distress before they escalate. In collaboration with a youth mental health charity,^1^ we assisted with the composition of song entitled ‘Bagels’, which was intended to reduce feelings of anxiety in youth. This song was developed with an established New Zealand pop entity (Benee) whose songs have been streamed over one billion times on the audio streaming service Spotify. The composition of the song was guided by the current authors, who referenced the accessible literature dedicated to the regulation of psychological states using music, and who directly worked with Benee and her team.

While subjective measures are most commonly found in the music literature, a growing number of studies using both subjective and objective measures, or objective measures in isolation, are being reported. Interestingly, while investigating music-induced emotion, Rickard (2004) found that skin conductance was a better indicator of emotion than self-report measures. In the current study we obtained both self-report (i.e., subjective) and electrophysiological (i.e., objective) data, which for the latter included measures of brain connectivity, skin conductance, and heart rate. All measurements were obtained while participants listen to Bagels and five other contemporary songs differing in affective character and spanning a range of genres popular with young people: ambient, pop, and metal. The main hypothesis of the current study is that subjective (i.e., state anxiety) and objective (i.e., brain connectivity, heart rate, skin conductance) measures of emotion will differ across the six songs, based on their affective characteristics. Specifically, we also predict that Bagels will be associated with lower state anxiety, lower skin conductance and heart rate, and lower alpha connectivity in the brain.

## Methods

### Participants

Thirty young adults [12 males (*M*_age_ = 23.3; *SD* = 2.14 years) and 18 females (*M*_age_ = 23.1; *SD* = 1.60 years)] served as volunteers for this study, with 27 reporting that they were right-handed. Due to equipment malfunction two participants were discarded from the analysis of skin conductance and heart, leaving 12 males (*M*_age_ = 23.25 years, *SD* = 2.14) and 16 females (*M*_age_ = 23 years, *SD* = 1.67). All participants reported good health, no history of hearing impairment, and were instructed not to consume caffeine or alcohol on the day of the experimental session. A small gift in the form of a supermarket voucher was offered to the participants at the terminus of the study, though this was not advertised beforehand. The study received approval from the [removed for anonymous review] Institutional Review Board.

### Song development

“Designer Music” consists of songs specially composed to manipulate the listener’s psychological and physiological states, for example, Marconi Union’s Weightless. As the brief for the Youthline project was to create a song that would prove both calming and popular with young adults, Bagels can be considered as part of the designer music genre. In order to appeal to youth, a New Zealand musician of international renown was selected: Stella Rose Bennett (stage name Benee: https://en.wikipedia.org/wiki/Benee). Along with her producer, Josh Fountain, Benee worked with the authors to create piece of music that utilized the findings reported in the scientific literature to have an anxiolytic effect, would fit with her brand, and appeal to youth. The selection of a local musician that would be familiar with New Zealand youth was also motivated by the observation that beyond the acoustic characteristics of a song, relaxation is also elicited by the associative nature of a song (Thaut and Davis, 1993).

In terms of creating a calming effect, the song was driven by a number of core recommendations found in the music therapy literature (e.g., Tan et al., 2012: Table 1; Elliott et al., 2011). The first was to avoid abrupt changes that could trigger a surprise response, as such ‘expectancy violations’ can induce autonomic arousal via an orientating response, which if recurring can elicit negative emotions. Instead, sustained melodic lines synonymous with relaxing music were preferred. Secondly, in line with Attention Restoration Theory, vocal contributions were constrained to Benee softly singing “You are not alone,” and a small uplifting voice-over toward the end of the song. Here, Benee’s speaking part centers on a story where the singer makes time to relax. Thirdly, as legato articulated music has been associated with tenderness and softness while staccato articulation reported to induce fear and anger (van der Zwaag et al., 2011), the composers were directed to transition notes smoothly and avoid silent gaps. Fourth, as fast tempos typically elicit reports of high arousal and energy, the composers were asked to maintain a low beats-per-minute, avoid percussion instruments that would be considered ‘sharp’, and employ simple rhythms. Fifth, based on numerous studies, a C major mode was selected in order to avoid inducing negative emotions such as sadness, which is typically evoked by minor modes. The completed song can be found at https://www.youtube.com/watch?v=ARzOSZqY_vo.

**Table 1:** Mean scores for affective and hedonic ratings across songs.

	Pleasantness	Arousal	Dominance	Likeability
(a) Bagels	7.34^b, c, d, *f*^ (1.35)	2.85^b,c,d,e^ (1.08)	3.95 ^b,c,d,e^ (1.64)	3.11^b,c,d,*f*^ (0.85)
(b) B.Y.O.B	3.10^a,d,e,*f*^ (1.68)	7.84 ^a,d,e,*f*^ (1.11)	8.32 ^a,d,e,*f*^ (1.19)	1.64^a,d,e,*f*^ (1.03)
(c) 5 Minutes Alone	2.82^a,d,e,*f*^ (1.67)	7.64 ^a,d,e,*f*^ (1.34)	8.16 ^a,d,e,*f*^ (1.25)	1.49^a,d,e,*f*^ (1.31)
(d) Schism	4.51^a, b, c, e, *f*^ (1.45)	5.98^a,b,c,*f*^ (1.04)	6.78 ^a,b,c,*f*^ (1.23)	2.48^a,b,c,e^ (0.98)
(e) Shape of You	7.11^b,c,d,f^ (1.19)	6.16^a,b,c,*f*^ (1.70)	6.88 ^a,b,c,*f*^ (1.57)	3.37^b,c,d,*f*^ (0.68)
(f) Weightless	6.19^a,b,c,d,e^ (1.44)	3.06^b,c,d,e^ (1.04)	3.86 ^b,c,d,e^ (1.67)	2.77^a,b,c,e^ (0.88)
*F*-statistic	51.837^***^	103.296^***^	62.076^***^	20.362^***^
η_p_^2^	0.658	0.793	0.697	0.430

Parentheses contain standard deviations. For any one mean score the superscript letters indicate Bonferroni-adjusted significant differences with the mean rating of other songs within the same column.

### Comparison songs

The song Bagels was compared to five other commercially available songs, four of which were chosen on merit of their contrasting psychophysical properties to Bagels. Each song lasted 250 s, and Adobe Audition was used to scale each song to an overall sound pressure level (SPL) of 65 dB SPL, with each song presented to the participants in stereo using 3 M E-A-RTONE with 3A inserts. The first comparison, Weightless by Marconi Union, was selected for benchmarking purpose, with its slow 60 beats-per-minute (BPM) rhythm, low dynamic range, and instruments that blend seamlessly, it has been described as ‘the most relaxing song in the world’ (Shepherd et al., 2023). As song familiarity has been related to music relaxation (Tan et al., 2012), we included a popular contemporary dance song, Shape of You by Ed Sherran, with a ‘build and release’ structure, a 96 BPM syncopated rhythm, and a minor key. Also in contrast to Bagels, the aggressive B.Y.O.B. by System of a Down is characterized by frequent dramatic tempo shifts (from 100 to 160 BPMs), switching between syncopated and distorted guitar riffs played in a minor key and a cleaner sounding chorus played in a major key. The menacing song 5 Minutes alone by Pantera is also in a minor key, but dynamically is more consistent than B.Y.O.B. with a steady but powerful drumbeat (122 BMP). Finally, to compare Bagels to a song that was inoffensive but with complex temporal shifts, Schism by Tool was selected. Schism is composed in the D Phrygian mode, and across time frequently changes time signatures (5/4, 7/8, and 6/8) between 60 and 75 BPM.

### Affective and hedonic measures

The ability of the six songs to impact emotions was assessed using the three-dimensional pleasant-arousal-dominance model (PAD: Bradley and Lang, 2000; Mehrabian and Russell, 1974), which was implemented according to Betella and Verschure (2016). Here, a temporal dynamics approach was employed, requiring participants to rate how pleasant (or unpleasant), how arousing (or calming), and how dominating (i.e., directing attention) the song-induced emotions were in real time using a ten-point sliding scale. In addition, a five-point rating scale was used to determine how likeable each song was, using a scale from 0 (0% likability) to 4 (100% likability). Note that a familiarity measure was eschewed as the song Bagels had yet to be released at the time of experimentation, though liking measures can be considered an adequate proxy measure of familiarity (Schäfer et al., 2013). Scale values were recorded every 500 ms, and as a song played the participants could change their ratings at will.

### Anxiety measures

Two versions of the State–Trait Anxiety Inventory (STAI: Spielberger et al., 1999) were presented: the full 40-item STAI was employed to measure trait anxiety, and for measurements of state anxiety after each song was played, participants completed the short-form STAI-6. The 40-item STAI is divided into two equal parts, measuring instantaneous anxiety (state anxiety) or anxiety more generally (trait anxiety), with only the latter half used as a baseline in the current study. For the STAI-6 participants were asked to make their ratings based on how they’re “feeling in the moment” (Groarke and Hogan, 2019). Responses were made using a four-point Likert Scale, with the STAI-6 items considered in the here-and-now (1 = Not At All, 2 = Somewhat, 3 = Moderately So, 4 = Very Much So), and trait anxiety items considered more generally (1 = Almost Never, 2 = Sometimes, 3 = Often, 4 = Almost Always).

### Brain activity

**Figure 1:**
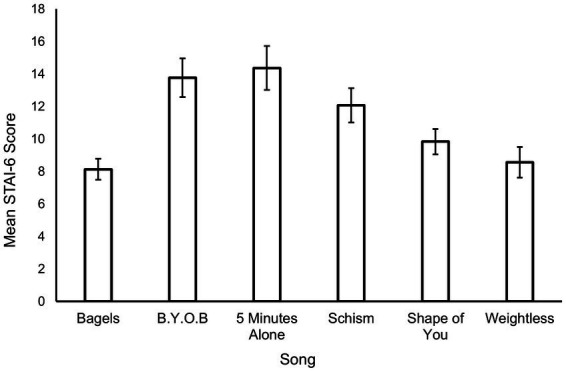
Mean STAI-6 state anxiety scores obtained following the termination of each song. Whiskers are 95% confidence intervals.

EEG datasets were obtained with a 64-channel Quik-Cap Neo Net cap and SynAmps 2/RT amplifiers (Figure 1). The data was processed in a Brain Imaging Data Structure (BIDS) format within the Python-based MNE software (RRID:SCR_008394) (version 1.3.1, Gramfort et al., 2014). EEG was recorded from 64 electrodes, down-sampling to 250 Hz from an initial sampling rate of 1,000 Hz. High-frequency signals were removed using a low-pass filter with a cut-off frequency of 100 Hz. 50 Hz line noise was eliminated using a notch filter. EEG data was virtually referenced using an average electrode referencing scheme. Noisy electrodes were interpolated with a spherical spline interpolation, while Independent Components Analysis (ICA) was employed to remove artifacts from the EEG signals by decomposing the EEG signals into 20 independent components. Each component was examined visually, and those related to eye movements, muscle activity, or other sources of artifacts were eliminated.

For each participant, the EEG data was converted into six epochs (one for each song) that were all 250 secinds long, resulting in 62,501 EEG time points per epoch. 56 electrodes were used for the connectivity analysis: FP1, FPZ, FP2, AF3, AF4, F7, F5, F3, F1, Fz, F2, F4, F6, F8, FC5, FC3, FC1, FCz, FC2, FC4, FC6, T7, C5, C3, C1, CZ, C2, C4, C6, T8, TP7, CP5, CP3, CP1, CPZ, CP2, CP4, CP6, TP8, P7, P5, P3, P1, PZ, P2, P4, P6, P8, PO7, PO3, POZ, PO4, PO8, O1, Oz, O2. EEG data processing and analysis were conducted using an iMac 3.6 GHz 10-Core Intel Core i9 and 64GB RAM. A cumulative 8 h of computation was done with MNE in Python (Gramfort et al., 2014).

### Autonomic measures

Skin conductance and cardiac data were acquired using a 24-bit Nexus-10 analog-to-digital converter (Mind Media BV) measuring eccrine sweat gland activity, used to calculate skin conductance level (SCL), and photoplethysmographic blood volume pulse (BVP) that was used to provide estimates of heart rate (HR). For SCL and BVP the data were obtained at sampling rates of 128 and 1,024 HZ, respectively. Change in Sympathetic Nervous System arousal is directly coupled to SCL, with greater SCL reflecting greater sympathetic activity. The recommendations of Boucsein et al. (2012) were followed when obtaining and analyzing SCL data. The BVP signal affords the estimation of HR using peak detection algorithms, with higher heart rates indicative of increasing sympathetic activation.

### Procedure

**Figure 2:**
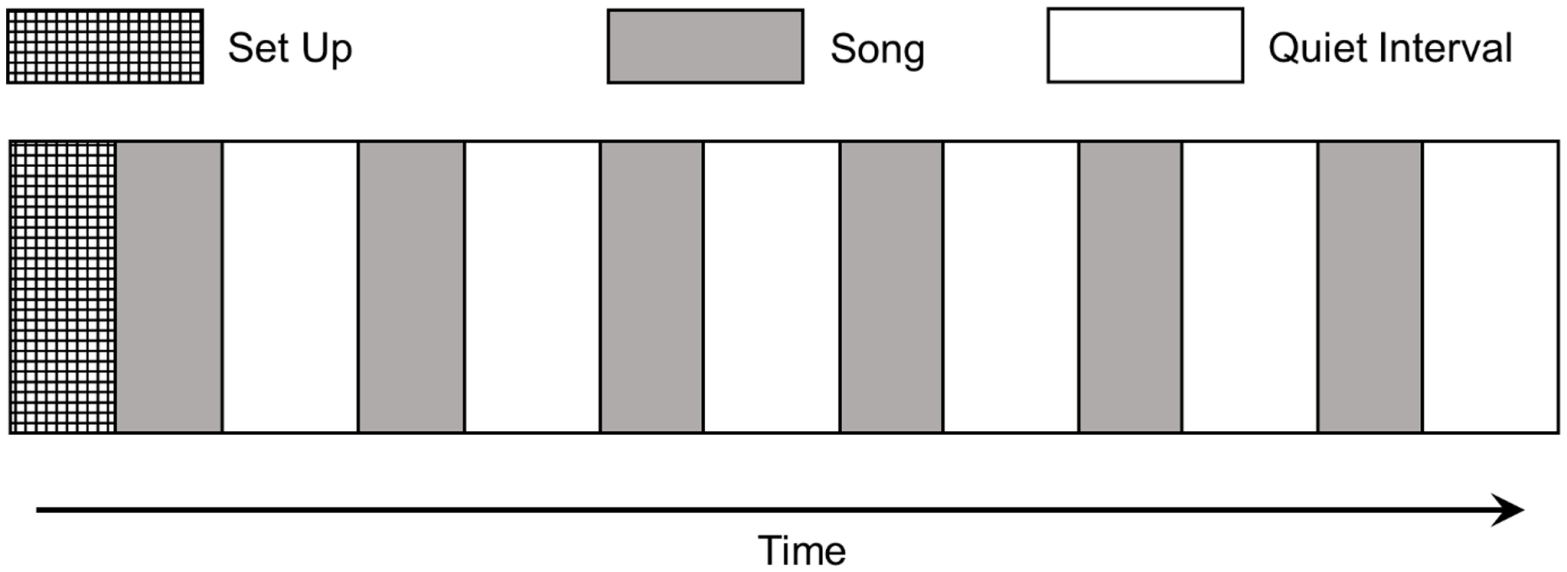
Experimental protocol. All songs were 250 s in duration and randomized across participants. Once initiated the procedure took approximately fifty minutes.

Participants attended a scheduled laboratory session and began the experiment by completing the full STAI in isolation. A single-subject design was utilized for the experiment itself, as per Figure 2. Data were collected from within a commercially obtained sound attenuating chamber containing a desk and chair, a color monitor, and a computer mouse. To begin, participants were comfortably seated. After exfoliation and cleansing using isopropyl alcohol, proprietary electrodes were applied to the volar surface of the medial phalanges on the middle and ring finger of the non-dominant hand in order to measure SCL. The BVP sensor was placed on the index finger, also on the non-dominant hand. Next, the EEG cap was positioned and set so that electrode impedances were below 10 kΩ.

As the electrophysiological sensors were attached, participants were briefed on what would happen during the session, and how and when to make the required responses. When set-up was complete and the participant reported that they were comfortable, the experiment began with an initial recording of two minutes silence. During this time the text on the monitor asked participants to relax and keep still. Immediately following the silent period, a randomized series of the six songs were presented, and as a song played the participants were asked to make affective judgments using the mouse and three sliders presented on the monitor, and also rate how much they liked the song. At the end of each song the affective and hedonic scales were removed and replaced with the STAI-6, which was displayed for 60 s. A three-minute rest-period followed in which participants were again asked to relax and keep still, providing a four-minute interval between the terminus of one song and the beginning of the next.

### Data analyses

#### Psychometric data

Affective and hedonic scores were collapsed across time as inspection revealed little change across time, justifying the use of a single mean score across the entire song interval. The mean affective, hedonic, and STAI-6 scores were then compared across the songs using repeated-measures analysis of variance (ANOVA) tests, with Bonferroni equalities applied *post-hoc* if the *F*-statistic was significant. The partial eta square (η_p_^2^) statistic is presented with ANOVA results to quantify effect size, with Cohen (1969) equating η_p_^2^ values of 0.0099, 0.0588, and 0.1379 to small, medium, and large effects, respectively.

#### Brain connectivity

Functional EEG connectivity was analyzed using the weighted Phase Lag Index (wPLI) for each song (Vinck et al., 2011). wPLI, calculated within the alpha (8–12 Hz) frequency band, is a measure of lagged synchronicity between pair-wise electrodes, denoting the phase differences of EEG signals between each electrode pair using the Hilbert transform. More specifically, for two time-series, x(t) and y(t), wPLI is derived from the imaginary part of their cross-spectrum, $S_{\mathrm{xy}}$:


$$\text{wPLI}=\frac{|E[I(S_{\mathrm{xy}})]|}{E[|I(S_{\mathrm{xy}})|]}$$

Here, E is the expected value and ℐ denotes the imaginary part of the cross spectrum as the imaginary values have less near zero-lag values and subsequently lower probability of volume conduction. wPLI ranges from 0 (no lagged synchronicity) to 1 (full lagged synchronicity).

In addition, three graph theory metrics provided EEG network properties, with repeated measures ANOVA used to test for EEG differences across the six songs. With six songs and three levels of graph theory metrics (local efficiency, node strength and betweenness centrality), this resulted in 30 separate Repeated Measures ANOVAs, with the graph theory metrics defined thus:

- Node strength is the total number of nodes (in this context, a node refers to EEG electrodes) and estimates a node’s connectedness to every other node in the network. Node strength is a metric for assessing a network’s general connectedness.
- Local efficiency measures connectivity between neighboring nodes in the network, calculated as the average of all pairs of a node’s neighbors’ inverse shortest path lengths. This metric measures communication effectiveness within a node’s local network.
- Betweenness centrality is defined as the shortest paths that run through a node on the network between all pairs of nodes and is utilized to compute a node’s betweenness centrality. A node’s importance in a network is determined by its betweenness centrality, or capacity to control how information is exchanged with other nodes, with more betweenness indicating greater importance.

#### Skin conductance and heart rate

Biotrace+, the proprietary software running the Nexus-10 hardware (Mind Media BV), analyzed the BVP signal to derive estimates of HR, provide measures of tonic SCL, and detect and eliminate artifacts. To better handle order effects and individual differences, the analytical approach first described by Miller and Ditto (1989) was adopted, whereby each individual’s electrophysiological dataset was transformed according to:


(1) $$\text{Percentage}\;(\%)\;\text{change}=(\begin{array}{l} \left(\mathrm{raw}\;\text{value}-\text{mean baseline value}\right)\\ /\text{mean baseline value} \end{array})\;x\;100.$$

Where the raw value is each recorded electrophysiological value, and the mean baseline was calculated from the 30 s of silence immediately preceding the song. Following transformation of the raw data a mean percentage change value across each song was obtained for each electrophysiological measure. These mean percentage change values were then compared across the six songs using a repeated-measures ANOVA, with significant *F*-values being followed by Bonferroni-adjusted *post-hoc* tests.

## Results

### Trait anxiety ratings

The full STAI was administered as a general screen for trait anxiety, where a score above 40 is conventionally used to indicate clinical anxiety levels (Emons et al., 2019). The mean trait anxiety score in the sample was 42.69 (*SD* = 9.85, *Min* = 26, *Max* = 70), indicating that on average the participants were at risk of clinical levels of anxiety.

### Affective and hedonic ratings

Descriptive statistics for mean responses to the three affective dimensions are displayed in Table 1, with the final column presenting overall likeability scores for each song. The theoretical minimum and maximum scores for the three affective dimensions are 1 and 10 respectively, while for the likeability ratings these are 0 and 4, respectively. Bagels, and its benchmark Weightless, were judged more pleasant and less arousing and dominating than the two harder metal songs (i.e., 5 Minutes Alone, B.Y.O.B). The superscript letters in Table 1 indicate the significant values obtained from post hoc testing of means across songs. Of note, Bagels had significantly higher mean pleasantness ratings than the other songs except for Shape of You, and was significantly less arousing than all songs except for Weightless.

### State anxiety ratings

The STAI-6 state anxiety scores collected following each song are displayed in Figure 1, accompanied by 95% confidence intervals. Inspection reveals that Bagels and Weightless had the lowest mean STAI-6 scores across participants, while two of the metal songs, B.Y.O.B and 5 Minutes Alone, had the highest mean STAI-6 scores. A repeated-measures ANOVA indicated that significant differences in STAI-6 scores existed across the six songs (*F*(5,136) = 36.228, *p* < 0.001, η_p_^2^ = 0.573). Subsequent Bonferroni post hoc tests showed that Bagels and Weightless did not differ significantly from each other, but significantly differed from all other songs except for Shape of You. Both 5 Minutes Alone and B.Y.O.B had significantly higher mean STAI-6 scores than all other songs though were not significantly different themselves, and any pairwise comparisons involving Schism were significantly different.

### Brain connectivity

**Figure 3:**
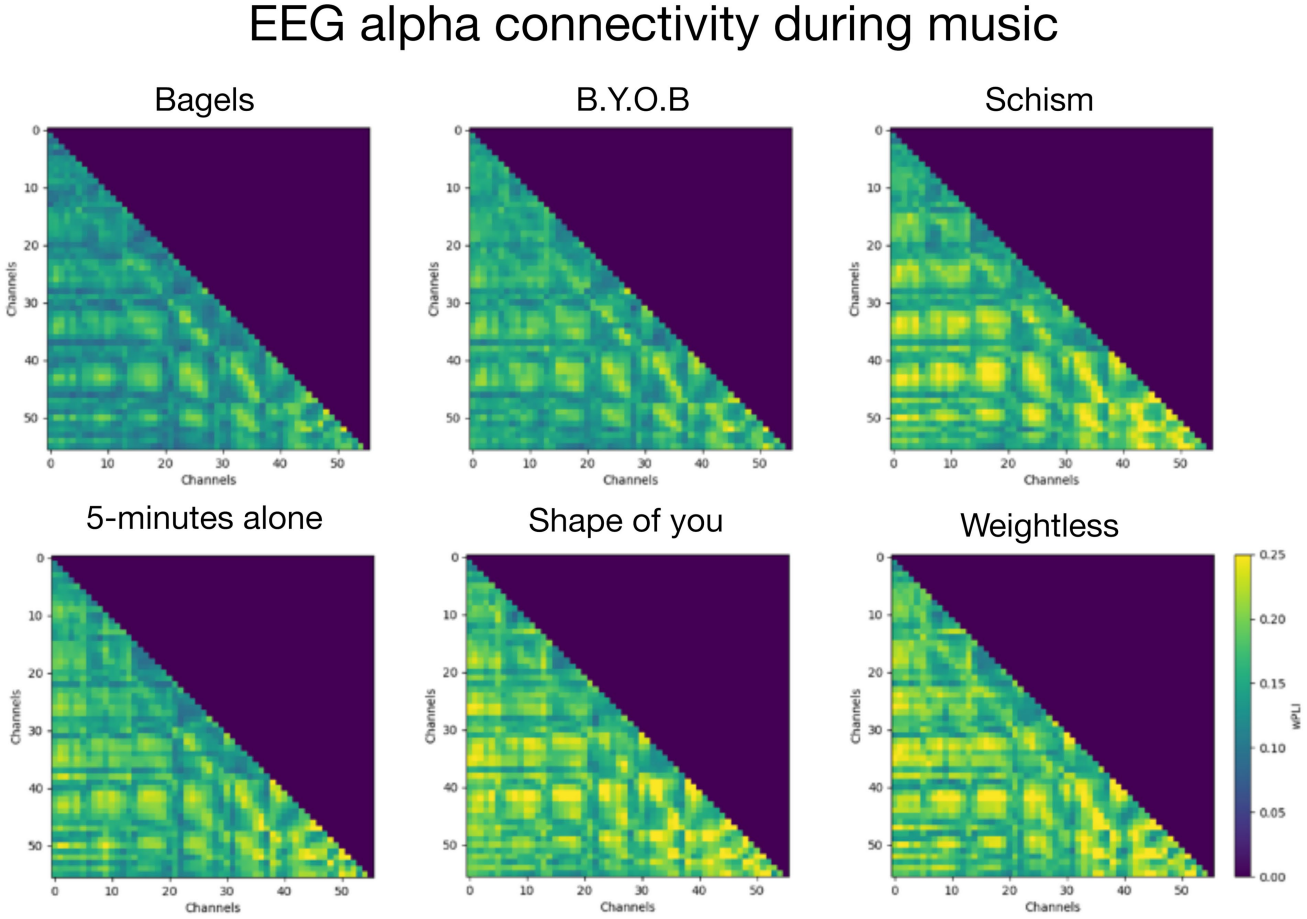
Connectivity matrices for all six songs. Colorbar is the same scale for all songs; the connectivity weights are represented as average wPLI values across all participants.

A repeated measures ANOVA was performed to quantify the statistical difference in network topology between the six songs. Node strength (“overall connectivity of nodes”) and local efficiency (“local connectedness of nodes”), but not betweenness centrality (“nodal network hubs”), had several EEG electrodes with a significant ANOVA after correcting for multiple comparisons (False Discovery Rate at *p* < 0.05). The largest effect size between songs was observed between the two ambient songs with reduced EEG alpha connectivity in the Bagels song compared to Weightless (see Figure 3). The significant electrodes were predominantly located in the frontal lobe for node strength and fronto-temporo-parietal lobes for local efficiency (see a complete overview of significant EEG electrodes in Table 2).

**Table 2:** Repeated measures ANOVA for EEG alpha connectivity metrics across 6 songs, corrected for multiple comparisons with False Discover Rate (FDR).

Node strength		Local efficiency	
FP1	*F*(5,145) = 5.45, *p* = 0.001	FP1	*F*(5,145) = 5.52, *p* = 0.002
FPZ	*F*(5,145) = 5.56, *p* = 0.002	FPZ	*F*(5,145) = 5.73, *p* = 0.001
FP2	*F*(5,145) = 5.14, *p* = 0.002	FP2	*F*(5,145) = 5.21, *p* = 0.005
AF3	*F*(5,145) = 4.48, *p* = 0.004	AF3	*F*(5,145) = 4.51, *p* = 0.005
AF4	*F*(5,145) = 4.78, *p* = 0.005	AF4	*F*(5,145) = 4.84, *p* = 0.005
F7	*F*(5,145) = 5.57, *p* < 0.001	F7	*F*(5,145) = 5.12, *p* = 0.001
F4	*F*(5,145) = 4.52, *p* = 0.007	F4	*F*(5,145) = 4.44, *p* = 0.007
F6	*F*(5,145) = 4.31, *p* = 0.009	F9	*F*(5,145) = 4.23, *p* = 0.009
F8	*F*(5,145) = 4.06, *p* = 0.009	FC6	*F*(5,145) = 4.05, *p* = 0.007
FC6	*F*(5,145) = 4.46, *p* = 0.005	TP7	*F*(5,145) = 4.88, *p* = 0.003
T7	*F*(5,145) = 3.81, *p* = 0.009	CP5	*F*(5,145) = 4.64, *p* = 0.002
TP7	*F*(5,145) = 4.21, *p* = 0.005	CP3	*F*(5,145) = 4.30, *p* = 0.006
TP8	*F*(5,145) = 3.83, *p* = 0.008	TP8	*F*(5,145) = 4.12, *p* = 0.009
P5	*F*(5,145) = 3.84, *p* = 0.008	P5	*F*(5, 145) = 5.73, *p <* 0.001
		P3	*F*(5,145) = 4.96, *p* = 0.002
		P6	*F*(5,145) = 4.40, *p* = 0.007
		P8	*F*(5,145) = 4.06, *p* = 0.007
		PO3	*F*(5,145) = 4.11, *p* = 0.006
		O1	*F*(5,145) = 4.53, *p* = 0.003

No significant electrodes for betweenness centrality.

### Autonomic response

**Figure 4:**
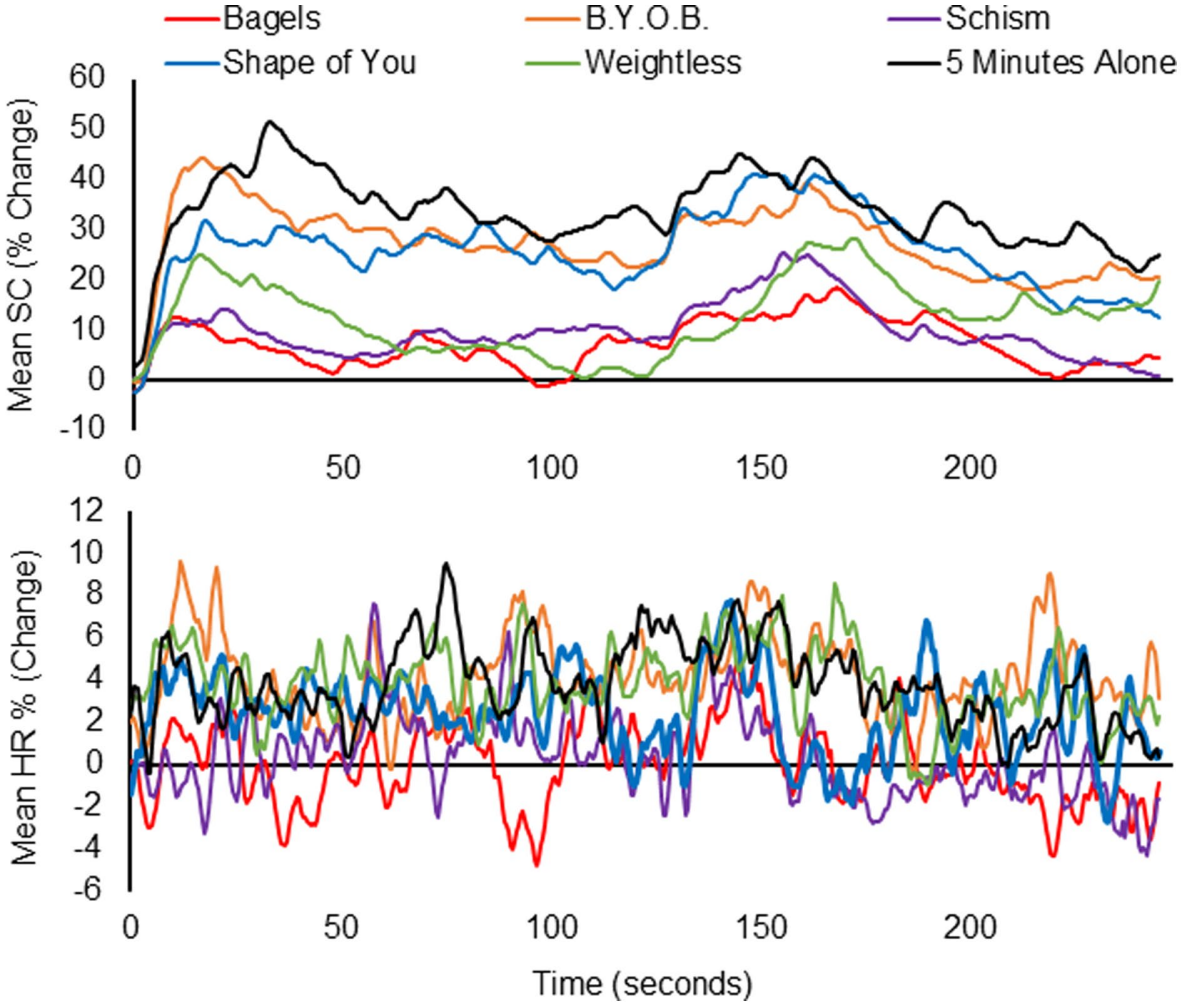
Pooled data showing mean percentage electrophysiological change as a function of time (seconds) for six songs. Electrophysiological measures are SC (top) and heart rate (bottom).

Figure 4 presents the pooled electrophysiological data for the six songs, with individual data transformed using Equation 1 prior to being averaging across participants. For Figure 4 (Top), and with reference to Table 1, the least arousing songs (Bagels, Weightless, Schism) changed SCL the least, while the three most arousing songs (5 Minutes Alone, B.Y.O.B., Shape of You) increased SCL the most. The trends with HR are less easily discerned by eye (Figure 4, middle), though the song rated most pleasant (Bagels) is associated with a decrease in HR. Interestingly, Weightless, which was designed to adjust tempo to systematically entrain the cardiac cycle, yielded an increase similar to the two songs associated with the highest arousal ratings: 5 Minutes Alone and B.Y.O.B. Further, Schism, which shifts time signatures during the chorus according to the Fibonacci sequence, was associated with a non-significant increase in HR.

Mean percentage change estimates (*re*: Equation 1) for the electrophysiological measures are displayed in Table 3, with one-samples t-statistics indicating if the mean was significantly different from 0 (i.e., no change between reference period and song). Repeated-measures ANOVA indicated that significant differences in SCL and HR existed across the six songs. For SCL the most liked song, Shape of You, was linked to greater sympathetic arousal than the two ambient songs (Bagels and Weightless) and to Schism. The two songs with the highest arousal ratings, 5 Minutes Alone and B.Y.O.B., had significantly higher percentage change means than Bagels, Schism, and Weightless. Turning to mean HR change, it was interesting to note significant differences between the two ambient songs (Bagels and Weightless), and that Weightless was statistical insignificant from the two most arousing songs, 5 Minutes Alone and B.Y.O.B.

**Table 3:** Mean electrophysiological percentage change values across the six songs.

	SCL	*t*	HR	*t*
(a) Bagels	3.22^b,c,e^ (8.29)	0.576	-0.068^b,c,*f*^ (4.23)	−0.043
(b) B.Y.O.B	27.64^a,d,*f*^ (29.69)	3.746^***^	4.145^a,d^ (4.14)	3.062^**^
(c) 5 Minutes Alone	25.80^a,d,*f*^ (28.37)	3.836^***^	3.788^a,d,^ (4.04)	3.507^**^
(d) Schism	9.83^b,c,e^ (22.65)	2.516^**^	0.314^b,c,*f*^ (3.76)	0.282
(e) Shape of You	33.88^a,d,*f*^ (28.14)	3.398^***^	2.373 (2.79)	3.282^**^
(f) Weightless	12.95^b,d,e^ (27.79)	2.262^*^	3.909^a,d^ (4.07)	2.566^*^
*F*-statistic	3.364^**^	-	2.224^*^	-
η_p_^2^	0.111	-	0.087	-

*p < 0.05, **p < 0.01, ***p < 0.001. Parentheses contain standard deviations. The superscript letters indicate significant differences with other songs for any one mean score. The columns labeled ‘t’ contain p-values obtained from single-samples t-tests in which the test statistic was set to zero.

## Discussion

The current study obtained subjective and objective measures from a set of songs that included Bagels, an original song designed to induce calm in young adults. Several findings from the current study are worthy of further discourse, centering on the effectiveness of the Bagels track. Secondary findings include a replication of the ability of music to induce emotion and regulate negative emotional states, and that even pre-selected (as opposed to self-selected) music can regulate emotion. These findings will be discussed with reference to anxiety scores measured in young adults, with half of the participants in the current study having STAI trait anxiety scores of forty or greater, indicating probable clinical levels of anxiety (Spielberger et al., 1999).

### Laboratory findings

Emotional regulation is the dominant function of music (Groarke and Hogan, 2019), whether being used to ‘wind down’ after a challenging day, or to arouse oneself prior to participating in some work or competition-related activity. The sounds encountered in everyday life can elicit emotional reactions, which in varying degrees can be experienced as pleasant, arousing, and dominant (Bradley and Lang, 2000). The PAD model adopted in the current study to measure the participant’s emotional responses to the six songs strongly emphasized the agency of music upon affective states. In terms of pleasantness scores, Bagels was rated higher than all other songs, indicating that of the six songs Bagels induced greater positive emotions such as happiness and contentment. Of note, Bagels was rated significantly more pleasant than its gold standard, Weightless, being rated approximately 20% more pleasant and thus suggestive of greater therapeutic potency. Bagels also obtained the lowest arousal ratings of the six songs, though for this measure it was not significantly different from Weightless, with the means of both songs indicating low levels of emotional activity and a relaxed and calm state (R Project for Statistical Computing: RRID:SCR_001905; LabView: RRID:SCR_014325).

In the music context, the final PAD dimension of dominance represents the degree to which a song evokes feelings of empowerment, control, or submission in the listener. For example, a song that is not directing attention allows a receiver to ruminate without interruption, and feel passive or reflective. Softer, delicate arrangements such as Bagels and Weightless would suggest lower dominance, as was confirmed in the dominance ratings collated in the current study. Further, the current study demonstrated that more aggressive and arousing songs (e.g., B.Y.O.B and 5 Minutes Alone) are associated with higher dominance ratings. Taken together, these findings reinforce the agency of music upon affective states, and specifically the potential of Bagels to be used as a song to alleviate over-arousal and discomfort through the induction of positive affective states (re: the Affect Infusion Model, Forgas, 1995). Furthermore, though music preference differs substantially across individuals, the current data suggests that affective ratings may be relatively independent of liking, as have other studies (Ritossa and Rickard, 2004). The implication here is that the self-selection of music may not be a pre-requisite for a therapeutic effect.

For the current set of songs, Bagels was associated with the lowest state anxiety scores, having mean scores significantly lower that the three metal songs (B.Y.O.B, 5 Minutes Alone, Schism), but statistically insignificant from Weightless and the pop-music track Shape of You (re: Figure 1). This is consistent with previous research indicating that music listening, as a form of music therapy, can reduce self-ratings of anxiety (Harney et al., 2023). However, some studies (e.g., Ribeiro et al., 2019) suggest that the positive emotions induced by music are transient and that the emotional effect of music may diminish within two minutes. Interestingly. after listening to Bagels and Weightless, participants reported lower average state anxiety ratings than their initial STAI trait scores. This suggests, in the music medicine context, that music may be better administered when anxiety presents itself, rather than being prescribed as a preventative medicine. The current study also indicates that to have an anxiolytic effect the consumer need not select the music, a topic finding that will be discussed further below.

Many playlists, artists and apps (e.g., https://www.calm.com; https://www.ipnos.com) aim to reduce anxiety and induce relaxation through meditation, music and ambient noise; however, few of these sonic interventions have been empirically tested, and fewer have undergone neuroscientific testing. This novel approach in the current study extends previous research on musical styles and demonstrates shifts in anxiety associated with music listening. Our study suggests that music can assist people in reducing subjective feelings of anxiety, and associated changes in brain connectivity. Although the EEG alpha activity literature is extensive and often with conflicting results (Miljevic et al., 2023), EEG alpha connectivity findings are still nascent. Despite this, previous research has shown that EEG alpha activity and connectivity may increase during music listening (Flores-Gutiérrez et al., 2009; Wu et al., 2012). Of note, the two songs with the lowest state anxiety ratings—the ambient songs, Bagels and Weightless—also had the most divergent EEG alpha connectivity patterns. Music listening affects multiple complex processes in the brain, including sensory perception/integration, cognition and emotion, and in the current context it is unlikely that the EEG findings solely relate to a reduction in anxiety. These findings are similar to previous fMRI work, which found bilateral and decreased activity with people listening to pleasant music (Koelsch et al., 2006). This activity, predominantly appearing in the limbic system, Heschl’s gyrus (auditory cortex), and inferior frontal structures, may be a pattern consistent with present study’s finding in the ambient song Bagels. Of note, EEG alpha connectivity was also lowest for the song Bagels, particularly in the brain’s frontal lobes.

Alongside participant reports of their affective states while listening to the song, objective measures of emotional response were also obtained. The current results are consistent with the findings of Labbé et al. (2007), who reported a greater reduction in skin conductance (SCL) for classical music compared to heavy metal music. Previous research found that Weightless decreased SCL faster than silence following a stress task (Shepherd et al., 2023), and for the current study, both Weightless and Bagels had the lowest SCL change following a period of silence. The lower electrophysiological arousal of Bagels and Weightless relative to B.Y.O.B and 5 Minutes Alone support Carpentier and Potter (2007) assertion that SCL has a proportional relationship with the pace of the music. An interesting finding between Bagels and its benchmark, Weightless, is that Bagels was associated with a small heart rate decrease across participants, while Weightless was associated with an increase sufficient to achieve statistical significance. A key difference between the songs is Bagel’s inclusion of a brief voice over, with Koelsch et al. (2011) suggesting the lyrics have the potential to comfort and calm.

### Self-selection of music

Much has been made of the assertion that music medicine should consist of self-selected rather than prescribed songs to be an effective intervention. The substance of the self-selection position is that preferred and familiar music elicits strong positive emotions, independent of the song’s affective properties (Jiang et al., 2016; Ritossa and Rickard, 2004), and gives the administrator a sense of control over their intervention. The opposing position that the psychophysical properties of a song, in particular tempo, dynamics, and melody, intrinsically drive the degree of relaxation, has also been expressed (e.g., Elliott et al., 2011). While the current study failed to measure familiarity, the data did suggest that the more a song was liked (i.e., preferred), the more anxiolytic potency it possessed. However, only a weak association between music likeability and both arousal (Rickard, 2004) and SCL (Kelley et al., 2014) has been reported. It may be that a liked song is played more, and that as familiarity with a song increases, so too does the possibility of habituation due to repeated exposure. Thus, further research is needed to determine the effects of frequently encountered songs upon emotional response and, in the intervention context, investigate whether individuals develop a tolerance to songs, and the impact of increasing music dose upon emotional states.

While the self-selection of music to self-regulate appears prima facie to be best practice, it assumes that the personal choices of the listener are indeed effective and optimal. In the context of adolescents and young adults, it can be argued that some popular genres unique to this cohort are neither calming nor relaxing (e.g., ‘Gangster Rap’ or ‘thrash metal’), and even though a song can be judged unpleasant, this is not equivalent to liking the song (Ritossa and Rickard, 2004). For example, the ‘Goth’ or ‘Emo’ genres characterized by sorrowful soundscapes and lyrics seeped in negative emotion may be preferred by young listeners as it reinforces their perceptions of low worth and self-efficacy. A similar argument could be made for subtypes of the heavy metal genre, which have been shown to increase levels of state anxiety, and there is evidence that some music genres may increase negative emotions in young people (e.g., Labbé et al., 2007; Thomson et al., 2014). Thus, the assumption that self-selected music is necessarily therapeutic for young adults needs further accreditation. In a recent meta-analysis, Harney et al. (2023) noted a lack of statistical significance when comparing the effects of participant vs. experimenter-selected music, which, along with the current findings, indicates that prescribed songs (e.g., Bagels or Weightless) may be of utility when attempting to reduce acute anxiety in young adults.

### Strengths and limitations

As with most laboratory-based research, several caveats should be considered when interpreting the findings. Firstly, even though participants sat in a comfortable reclining chair, the experimental context limits ecology validity. Next, a limited range of songs were used, none of which could be considered classical music, which has been promoted as a benchmark genre (Adiasto et al., 2022). Instead, music considered more appropriate to young adults was selected in the current study. A formal ‘silent’ condition was likewise omitted, even though previous research into the anxiolytic effects of music primarily utilizes silence as a comparison (Panteleeva et al., 2018). Instead, in the current study the reference for the percentage change in electrophysiological activity was the thirty seconds of silence prior to a song playing. Though the comparison changed from song-to-song, it is seen as a superior approach to control for confounding variables such as order effects or electrophysiological habitation (Miller and Ditto, 1989). Finally, the modest sample size limits the generalisability of the current findings, however, it could be argued that the 28–30 participants were sufficient in number given the repeated measures design and the measures being obtained.

Considering the current design further, the single session approach adopted in the current study was chosen to minimize participant discomfort and attrition, but was vulnerable to carryover effects despite the randomization of song order across participants. One solution is to use a multisession approach in which participants listen to a single song during a single session, though the drawback of this approach is the potential for increased variability in participant vigilance or mood states across sessions. Furthermore, in relation to experimental design, the use of a double-blind approach may have reduced the potential bias arising from participants expectations, and in the current study we attempted to minimize bias by promoting the study as an investigation into the neural correlates of music. In terms of the sample profile, recruitment focused on age while attempting to balance gender, but did not attempt to control for cultural differences. Likewise, it would have been interesting to investigate if prior musical training had an effect on the affective response to the songs, as research indicates that while music is equally enjoyed by musicians and non-musicians alike, it has been reported that musicians are more analytical and objective when judging the merits of the songs (Wolfe et al., 2002).

Finally, the limitations inherent in EEG recordings, particularly its low spatial resolution, complicates the attribution of specific cognitive or emotional processes to individual scalp electrodes. While the significant differences were primarily observed in frontal and temporal regions - areas commonly associated with emotional and auditory processing - we caution against overinterpretation of the reported EEG data. Future studies incorporating source localization techniques or multimodal imaging are needed to more precisely map these network dynamics to underlying neural substrates.

### Implications and future directions

These findings reinforce a handful of other studies indicating that commercially available songs can reduce state anxiety, and add to the body of literature by reporting both subjective and objective measures from young adults. Music medicine, involving music listening as opposed to music making, is a relatively easy and cost-effective means to target maladaptive emotions due to the ease of accessibility of songs in the digital age. As an alternative treatment for anxiety, music avoids both the side-effects associated with anxiolytic medication and the financial and time costs associated with therapy, thereby increasing treatment adherence. A further advantage is that individuals have some control over the dose they receive and when and where it is administered, without frequent in-person consultations with a practitioner. Indeed, an individual managing their intervention may increase perceptions of self-efficacy, with low self-efficacy linked to dysfunctional ruminations on coping with challenging situations and anxiety (Ekizoglu and Ozcinar, 2010).

An interesting finding emerging from this study is that even music not selected by an individual can have the agency to calm and increase parasympathetic dominance. Specifically, both the Bagels and Weightless song were associated with lower state anxiety ratings and electrophysiological arousal than the songs from the heavy metal genre. The implication is that the musical features of song, such as tempo and key, may be as effective in reducing state anxiety as song familiarly or even preference (Elliott et al., 2011). This finding may have implications for music played in public spaces. While it is unlikely that each-and-every individual will respond uniformly to songs such as Bagels and Weightless, they may still be helpful in healthcare or educational environments to elicit positive emotions in individuals experiencing acute anxiety. Likewise, environments fabricated to induce excitement such as sports arenas could benefit from high arousal and low valance songs such as B.Y.O.B.

There is no ‘silent’ in music medicine, and the current thinking that silence is the gold standard in stress reduction and emotional regulation arguably needs more consideration. Firstly, the evidence for the superiority of silence over music is wanting (Shepherd et al., 2023; Groarke and Hogan, 2019), as silent environments may induce negative ruminations and increase cognitive arousal (Lui and Grunberg, 2017). Secondly, silence may not always be achievable in urban and suburban environments, or individuals may simply prefer to listen to sound over quiet. Thus, when noise masks silence, music can mask the noise. Future research directly comparing music medicine to silence would be informative (Harney et al., 2023), as would confirming the status of silence as the gold standard reference condition in research. However, while music medicine harbors promise as a means to regulate negative emotions in young adults, further research is needed to maximize its effectiveness (Tan et al., 2012), including longitudinal studies that are performed in real-world settings. In relation to the current study, future longitudinal research incorporating a larger sample of young adults could assess the persistence and sustainability of Bagel’s anxiolytic effects.

## Conclusion

In conclusion, the use of music in stress management shows promise, and music provides a potentially easy, appealing, and cost-efficient anxiolytic that people can administer wherever they are (McFerran et al., 2018). The current findings reinforce suggestions from the literature arguing that careful music creation and selection are key, and that some styles of music intrinsically increase arousal and feelings of anxiety while others do not (Thomson et al., 2014). In this study we report on the development and evaluation of a song specifically designed to relax young adults, Bagels, which performed as well or even better than a gold standard (i.e., Weightless), and was more liked. We welcome the inclusion of Bagels in future studies into music and psychological well-being, and assessments of its potential as an anxiolytic.

## Data Availability

The raw data supporting the conclusions of this article will be made available by the authors, without undue reservation.

## Footnotes

^1^ www.youthline.co.nz
